# Relationship of objectively measured physical activity and sedentary behaviour with health-related quality of life among breast cancer survivors

**DOI:** 10.1186/s12955-020-01478-x

**Published:** 2020-07-10

**Authors:** Ali Nurnazahiah, Mohd Razif Shahril, Zakarai Nor Syamimi, Aryati Aryati, Suhaina Sulaiman, Pei Lin Lua

**Affiliations:** 1 Faculty of Health Sciences, Universiti Sultan Zainal Abidin, Gong Badak Campus, 21300 Kuala Nerus, Terengganu Malaysia; 2 Centre for Healthy Ageing and Wellness (H-CARE), Faculty of Health Sciences, Universiti Kebangsaan Malaysia, Jalan Raja Muda Abdul Aziz, 50300 Kuala Lumpur, Malaysia; 3 Faculty of Pharmacy, Universiti Sultan Zainal Abidin, Besut Campus, 22200 Besut, Terengganu Malaysia

**Keywords:** Physical activity, Sedentary behaviour, Health-related quality of life, Breast cancer survivors

## Abstract

**Background:**

Lack of physical activity throughout one’s lifetime has been associated with obesity and it is also an important risk factor of breast cancer. This study aimed to determine the relationship between objectively measured physical activity and sedentary behaviour with health-related quality of life (HRQoL) among breast cancer survivors in the East Coast region of Peninsular Malaysia.

**Methods:**

A cross-sectional study involving 83 breast cancer survivors was carried out in two main government referral hospitals in the region. Participants wore the ActivPAL3™ microdevice physical activity monitor for seven consecutive days. The validated European Organization for Research and Treatment of Cancer Quality of Life Questionnaires (EORTC QLQ–C30) and Breast Cancer Supplementary Measure (EORTC QLQ-BR23) were used to measure their HRQoL. Multiple linear regression analysis was conducted to determine the relationship between objectively measured physical activity and sedentary behaviour with HRQoL.

**Results:**

Longer time spent on moderate to vigorous physical activity (MVPA) was significantly associated with an improvement of HRQoL (*p* = 0.039) whereas longer time spent on sedentary behaviour significantly reduced the functioning score (*p* = 0.005). In addition, prolonged sedentary bouts were also significantly associated with better body image that led to improved HRQoL (*p* = 0.013).

**Conclusions:**

The study findings suggest that an increase in the time spent on MVPA was associated with improved HRQoL while sedentary behaviour was associated with poorer HRQoL among breast cancer survivors. Thus, it is essential to displace sedentary behaviour with MVPA to improve the quality of life of breast cancer survivors.

## Introduction

With the early detection and improved treatment, the number of breast cancer survivors is increasing in Asia. Many of the patients who were diagnosed with early-stage breast cancer had 5-year survival rates exceeding 90% [1]. Nonetheless, the survival rate is lower among Malaysian breast cancer survivors compared to the survivors in the Western countries with more than 80% of the total survival rate [2]. It is common for breast cancer survivors to experience physical and emotional challenges during their treatment and recovery phases. These challenges may compromise their abilities to sustain independent lifestyles and may also decrease their health-related quality of life (HRQoL) [3]. They are also at a higher risk of developing fatigue and pain as well as psychological distress such as depression and fear of cancer recurrence. All these may result in in compromised HRQoL [4]. In a recent meta-analysis, breast cancer survivors in Asia were found to have poorer HRQoL than the general population [1]. However, it can be improved over time while having healthier behaviours.

Physical activity has been consistently found to be a crucial element in the therapy of various chronic diseases as it has been proven to improve HRQoL and reduce mortality [5]. Physical activity is also a potent non-pharmacological therapy among cancer survivors [6]. Being physically active during and after breast cancer treatment has been shown to produce beneficial health outcomes [7], such as decreasing the risk of recurrence and death, as well as reducing the anxiety associated with cancer recurrence [8]. There is a growing body of evidence to support that an increased level of physical activity after breast cancer diagnosis improved the survivors’ HRQoL and self-esteem while reducing their psychosocial distress [9]. It has also been reported that increased levels of low-intensity physical activity led to better HRQoL among cancer survivors [10].

On the contrary, physical inactivity and sedentary behaviour are associated with poorer health consequences among breast cancer survivors and they were also a barrier towards the improvement of HRQoL. For many breast cancer survivors, besides failing to achieve the recommended daily physical activity, they also spend the majority of their time on sedentary behaviours that lead to negative health effects [11]. An earlier study demonstrated that less than 30% of individuals diagnosed with cancer achieved the recommended level of physical activity [12]. Based on guidelines for physical activity in cancer survivors by the American College of Sports Medicine, if they fail to achieve physical activity recommendation, they should at least try to prevent physical inactivity and be as physically active as they can [13].

Hence, it is crucial to explore the effects of physical activity and sedentary behaviour on HRQoL among breast cancer survivors. Numerous studies had attempted to evaluate the relationship between physical activity [9, 12] and sedentary behaviour [14] with HRQoL among breast cancer survivors. Most of the studies found a positive relationship between physical activity and HRQoL as well as a negative relationship between sedentary behaviour and HRQoL among breast cancer survivors. However, most of these studies were carried out in the Western countries with limited evidence from the Asian countries. Furthermore, very few of these studies assessed physical activity and sedentary behaviour by using objective measurement. It is crucial to obtain more information about the effect of objectively measured physical activity and sedentary behaviour on HRQoL among the non-Western population. ActivPAL has been used to differentiate between sitting or lying, standing, and stepping activities and it has been validated for use among adults [15, 16]. Additionally, a recent study reported that ActivPAL has a high accuracy to estimate time spent in the physical activity intensity category by using step frequency to distinguish low intensity physical activity and MVPA [17]. Therefore, this study aimed to determine the relationship between objectively measured physical activity and sedentary behaviour with HRQoL among Malaysian breast cancer survivors.

## Methods

### Participants

A cross-sectional study was conducted from November 2015 to February 2016 among breast cancer survivors who have completed their treatment in the East Coast of Peninsular Malaysia. The participants were recruited at the surgical outpatient clinics at Hospital Sultanah Nur Zahirah, Kuala Terengganu, Terengganu and Hospital Raja Perempuan Zainab II, Kota Bharu, Kelantan. Ethical approval was obtained from the Medical Research and Ethics Committee (MREC), Ministry of Health Malaysia [Registration: NMRR-14-1618-23,717 (IRR)].

A total of 83 participants were selected through purposive sampling. The inclusion criteria were breast cancer survivors who were at least 18 years old at the time of diagnosis, diagnosed with cancer of stages I to III, completed conventional treatments (surgery, chemotherapy, and/or radiotherapy) at least 6 months prior to recruitment, and free from secondary cancer or breast cancer recurrence. All subjects were given an information sheet with details about the study purpose, research importance, and ethical issues. Only participants who agreed to join and provided written consent were included in the study.

### Objectively measured physical activity and sedentary behaviour

The time spent on sedentary behaviour and moderate-to-vigorous physical activity (MVPA) during the waking hours were measured using the ActivPAL3™ microdevice (PAL Technologies Ltd., Glasgow, UK). The device was attached on the mid-right thigh. The thigh position was used to generate information on the time spent in different body positions (horizontal = lying or sitting; vertical = standing or stepping). The start and stop time of each participant’s bout (or event) of lying or sitting, standing, and stepping were recorded by the device. The participants needed to wear the device for 24 h and seven consecutive days. The time spent sitting or lying during the waking hours was measured as sedentary behaviour [18] whereas the sum of time spent on standing or stepping was measured as physical activity. The percentage of the sedentary behaviour spent per day (sedentary behaviour/ waking hours) was calculated for each participant. On the other hand, quality control checks were applied to identify non-compliance during the wearing period (i.e., ≥10 h/day of wear) or other problems with the data (i.e., monitor malfunction or the device being attached upside down). Only valid days with at least 10 h of wearing would be included to produce the daily average of the relevant parameters [19].

As each participant had different wake-up times and bedtimes, an automated algorithm was used to recognise the wake-up and bedtimes of each respondent for several days. The algorithm offered a high accuracy in determining the waking time compared to self-reporting by the participant because it used the number and duration of sedentary periods to recognise bedtimes as well as the number and duration of active periods (standing or stepping) to identify wake times [19]. The number of sedentary breaks during the waking hours was known as the transitions from sitting or lying to standing or stepping [20]. With that, the mean number of breaks per day was calculated. Sedentary time that occurred in an uninterrupted period of at least 30 min was considered as a prolonged sedentary bout and the mean number of prolonged sedentary bouts during waking hours per day was calculated. The average bout duration was assessed by dividing the total sedentary time with the total number of sedentary bouts. Minutes with a step frequency of more than 110 steps/minute were categorised as MVPA [21].

### Health-related quality of life (HRQoL)

The validated European Organization for Research and Treatment of Cancer Quality of Life Questionnaires (EORTC QLQ–C30) which had been translated into Malay [22] was used to assess HRQoL among breast cancer survivors. The validity and reliability of this instrument in assessing the HRQoL of cancer patients in diverse study settings have been proven in an earlier study [23]. It was designed to be cancer-specific, multi-dimensional in structure, suitable for various cultural backgrounds, appropriate to use with supplementary site or treatment specific modules, and suitable for self-administration. The questionnaire consists of 30 cancer-specific questions to evaluate the HRQoL of cancer patients in four different domains, i.e. global health, functioning, symptoms, and financial implications of the diseases. Breast Cancer Supplementary Measure (EORTC QLQ-BR23) was also used in conjunction with the parent EORTC QLQ-C30 to assess HRQoL of breast cancer patients. The EORTC QLQ-BR23 is made up of 23 questions of functional scale and symptom scale.

According to the EORTC scoring manual, all of the scores from 1 to 4 or from 1 to 7 were changed to a score from 0 to 100 [23]. The raw scores were linearly changed to get standard scores in the range of 0–100 for each of the scales and single items. A high scale score indicated a higher response level. Therefore, a high score for a functional scale represented a high or healthy level of functioning and a high score for the global health status showed a better HRQoL. Additionally, a greater level of symptomology/ problems was represented by a high score for a symptom scale.

### Statistical analysis

Descriptive characteristics of the study sample are summarised as mean and standard deviation (SD) or as numbers and percentages. To examine the relationship of objectively measured physical activity and sedentary behaviour with HRQoL, multiple linear regression adjusted for age, body mass index (BMI), cancer stage at diagnosis, duration since diagnosis, education level, and working status was performed. Statistical significance was taken as *p*-value less than 0.05. All the analyses were conducted with IBM SPSS version 22.0 (IBM, Armonk, NY, USA) and met the assumptions for regression prior to analysis. There were no missing data in this study for all variables.

## Results

As shown in Table 1, the study participants had a long period of survivorship in which 73.5% of them survived for more than 5 years after been diagnosed. Most of them were diagnosed as stage II cancer (56.6%). On average, breast cancer survivors spent 58.4% of their waking hour on sitting/lying, 31.1% on standing and 10.5% on stepping. Within the stepping time, participants in this study spent an average of 27.95 min/day in MVPA.

**Table 1:** Characteristics of Breast Cancer Survivors (*n* = 83)

Characteristics	Mean (SD)	n	%
Age (years)	52.8 (7.8)		
BMI (kg/m^2^)	27.8 (4.8)		
Underweight		2	2.4
Normal		18	21.7
Overweight		38	45.8
Obese		25	30.1
Education level			
Primary		5	6.0
Secondary		52	62.7
College/University		26	31.3
Working status			
Working		43	51.8
Not working		40	48.2
Duration since diagnosis (years)	6.84 (4.13)		
< 5-year		22	26.5
> 5-year		61	73.5
Cancer stage			
Stage I		13	15.7
Stage II		47	56.6
Stage III		23	27.7
Treatment			
Surgery		83	100.0
Chemotherapy		83	100.0
Radiotherapy		73	88.0
No. of valid days	6.21 (1.24)		
Waking hours (hour/day)	17.85 (1.16)		
Standing (hour/day)	5.55 (1.71)		
Stepping (hour/day)	1.87 (0.63)		
Step counts (count/day)	7882.47 (2832.59)		
MVPA (min/day)	27.95 (17.96)		
Sedentary time (hour/day)	10.42 (1.79)		
Shorter sedentary bouts [< 30 min] (count/day)	51.19 (8.85)		
Prolonged sedentary bouts [≥30 min] (count/day)	9.47 (3.93)		
Mean length of sedentary bouts duration (min)	9.89 (0.90)		
Sedentary breaks (count/day)	51.55 (12.66)		

*SD* Standard deviation, *MVPA* Moderate to vigorous physical activity

The results of the multiple regression analysis on the relationship of objectively measured physical activity and sedentary behaviour with HRQoL before and after adjusting for age, BMI, cancer stage at diagnosis, duration since diagnosis, education level, occupation, and waking hours are presented in Table 2. MVPA (*p* = 0.039) was the only significant variable for global health status. On the other hand, sedentary time was significantly associated with functional scores (*p* = 0.005). Increased time spent on sedentary behaviour was found to significantly decrease the HRQoL of breast cancer survivors.

**Table 2:** Relationship of Objectively Measured Physical Activity and Sedentary Behaviour with HRQoL (EORTC QLQ-C30)

EORTC QLQ-C30						
	Global Health Status		Functional Scores		Symptom Scores	
	Regression Coefficient (β) (95% CI)	***p***-value	Regression Coefficient (β) (95% CI)	***p***-value	Regression Coefficient (β) (95% CI)	***p***-value
Sitting/lying (hour/day)						
Unadjustedª	0.375 (−1.463, 2.214)	0.686	−1.865 (−2.937, − 0.792)	0.001*	1.614 (0.535, 2.693)	0.004*
Adjusted^b^	1.327 (− 0.711, 3.366)	0.198	− 1.705 (− 2.872, − 0.539)	0.005**	0.139 (− 0.035, 0.314)	0.115
Standing (hour/day)						
Unadjustedª	0.030 (− 1.897, 1.956)	0.976	1.043 (− 0.137, 2.223)	0.083	−1.230 (−2.388, − 0.072)	0.038*
Adjusted^d^	1.732 (−5.420, 8.884)	0.631	−1.680 (−5.663, 2.303)	0.403	1.234 (−3.101, 5.570)	0.572
Stepping (hour/ day)						
Unadjustedª	−1.471 (−6.680, 3.738)	0.576	4.575 (1.478, 7.672)	0.004*	−3.621 (−6.742, − 0.500)	0.024*
Adjusted^d^	−1.713 (−8.866, 5.440)	0.634	1.628 (− 2.357, 5.613)	0.418	−1.172 (−5.510, 3.166)	0.592
Low intensity stepping (min/day)						
Unadjustedª	−0.083 (− 0.190, 0.024)	0.125	0.065 (− 0.001, 0.132)	0.052	− 0.064 (− 0.129, 0.001)	0.054
Adjusted^d^	− 0.128 (− 0.261, 0.006)	0.061	0.010 (− 0.066, 0.087)	0.785	0.001 (− 0.080, 0.081)	0.993
MVPA (min/day)						
Unadjustedª	0.129 (−0.052, 0.309)	0.160	0.150 (0.041, 0.260)	0.008*	−0.084 (− 0.195, 0.028)	0.138
Adjusted^d^	0.218 (0.011, 0.425)	0.039**	0.060 (−0.057, 0.178)	0.310	−0.006 (− 0.131, 0.119)	0.921
Step counts (count/day)						
Unadjustedª	0.001 (−0.001, 0.002)	0.656	0.002 (0.001, 0.002)	0.032*	−0.001 (− 0.002, 0.001)	0.106
Adjusted^d^	0.001 (−0.002, 0.002)	0.538	0.001 (−0.001, 0.002)	0.776	0.001 (−0.001, 0.002)	0.860
Shorter sedentary bouts (count/day)						
Unadjustedª	0.133 (−0.237, 0.503)	0.475	−0.141 (− 0.370, 0.089)	0.226	0.180 (− 0.046, 0.405)	0.117
Adjusted^c^	0.053 (−0.424, 0.531)	0.824	0.104 (−0.164, 0.371)	0.442	0.028 (−0.261, 0.316)	0.850
Prolonged sedentary bouts (count/day)						
Unadjustedª	0.207 (−0.628, 1.042)	0.623	−0.601 (−1.106, − 0.097)	0.020*	0.361 (− 0.149, 0.870)	0.163
Adjusted^c^	0.487 (−0.764, 1.739)	0.440	−0.138 (− 0.849, 0.572)	0.699	− 0.329 (− 1.085, 0.427)	0.388
Sedentary breaks (count/day)						
Unadjustedª	0.136 (−0.117, 0.389)	0.288	0.148 (0.004, 0.292)	0.045*	−0.107 (− 0.250,0.035)	0.138
Adjusted^c^	−0.155 (− 0.448, 0.138)	0.294	− 0.038 (− 0.206, 0.131)	0.658	0.142 (− 0.034, 0.317)	0.111
EE (MET.h)						
Unadjustedª	0.382 (−1.293, 2.057)	0.651	0.382 (−1.293, 2.057)	0.651	0.810 (−0.222, 1.841)	0.122
Adjusted^d^	−0.108 (−2.650, 2.435)	0.933	0.155 (−1.265, 1.574)	0.829	0.569 (−0.917, 2.055)	0.448

ª Crude regression coefficient by simple linear regression, ^b^ Adjusted regression coefficient by multiple linear regression for BMI, age, cancer stage at diagnosis, duration since diagnosis, education level, working status, waking hours and MVPA. ^c^ Adjusted regression coefficient by multiple linear regression for BMI, age, cancer stage at diagnosis, duration since diagnosis, education level, working status, waking hours, sedentary time and MVPA. ^d^ Adjusted regression coefficient by multiple linear regression for BMI, age, cancer stage at diagnosis, duration since diagnosis, education level, working status, waking hours and sedentary time. * Significant *p*-value (< 0.05) for unadjusted variables. ** Significant *p*-value (< 0.05) for adjusted variables. *MVPA* Moderate to vigorous physical activity. *EE (MET.h)* Energy expenditure (Metabolic Equivalent of Task hours)

The relationship of objectively measured physical activity and sedentary behaviours with HRQoL according to breast cancer supplementary measure is shown in Table 3. Only prolonged sedentary bout was found to be significantly associated with functional scores after adjusted for various confounders. Increased time spent on prolonged sedentary behaviour was associated with improved functional scores (*p* = 0.013).

**Table 3:** Relationship of Objectively Measured Physical Activity and Sedentary Behaviour with HRQoL (EORTC QLQ-BR23)

	EORTC QLQ-BR23			
	Functional Scores		Symptom Scores	
	Regression Coefficient (β) (95% CI)	***p***-value	Regression Coefficient (β) (95% CI)	***p***-value
Sitting/lying (hour/day)				
Unadjustedª	0.835 (−1.581, 3.252)	0.494	0.252 (− 0.699, 1.204)	0.599
Adjusted^b^	− 0.240 (−2.695, 2.215)	0.846	− 0.066 (− 0.176, 0.044)	0.233
Standing (hour/day)				
Unadjustedª	0.628 (−1.905, 3.160)	0.623	− 0.224 (− 1.220, 0.772)	0.656
Adjusted^d^	2.700 (− 0.651, 11.051)	0.521	1.066 (−2.661, 4.793)	0.570
Stepping (hour/day)				
Unadjustedª	−1.685 (−8.547, 5.178)	0.627	−1.594 (−4.274, 1.085)	0.240
Adjusted^d^	−2.687 (−11.039, 5.666)	0.523	−1.054 (− 4.782, 2.674)	0.575
Low intensity stepping (min/day)				
Unadjustedª	−0.016 (− 0.15, 0.126)	0.820	− 0.019 (− 0.075, 0.037)	0.495
Adjusted^d^	− 0.051 (− 0.212, 0.109)	0.525	0.001 (− 0.069, 0.071)	0.978
MVPA (min/day)				
Unadjustedª	−0.077 (− 0.318, 0.163)	0.523	− 0.063 (− 0.156, 0.031)	0.188
Adjusted^d^	− 0.040 (− 0.288, 0.209)	0.752	−0.066 (− 0.176, 0.044)	0.233
Step counts (count/day)				
Unadjustedª	−0.001 (− 0.002, 0.001)	0.317	0.001 (− 0.001, 0.002)	0.378
Adjusted^d^	−0.001 (− 0.003, 0.001)	0.245	0.001 (− 0.001, 0.002)	0.753
Shorter sedentary bouts (count/day)				
Unadjustedª	−0.206 (0–.693, 0.280)	0.401	0.016 (−0.176, 0.208)	0.869
Adjusted^c^	0.014 (−0.561, 0.569)	0.961	−0.011 (− 0.265, 0.234)	0.931
Prolonged sedentary bouts (count/day)				
Unadjustedª	1.499 (0.450, 2.5490	0.006*	−0.058 (− 0.491, 0.374)	0.789
Adjusted^c^	1.859 (0.413, 3.306)	0.013**	−0.261 (− 0.927, 0.405)	0.437
Sedentary breaks (count/day)				
Unadjustedª	−0.058 (− 0.396, 0.281)	0.736	− 0.085 (− 0.210, 0.039)	0.175
Adjusted^c^	− 0.227 (− 0.579, 0.124)	0.201	0.040 (− 0.117, 0.197)	0.616
EE (MET.h)				
Unadjustedª	0.222 (−1.985, 2.429)	0.842	−0.277 (− 1.143, 0.589)	0.526
Adjusted^d^	−1.479 (−4.431, 1.474)	0.321	0.418 (−0.904, 1.741)	0.197

ª Crude regression coefficient by simple linear regression, ^b^ Adjusted regression coefficient by multiple linear regression for age, BMI, cancer stage at diagnosis, duration since diagnosis, education level, working status, waking hours and MVPA. ^c^ Adjusted regression coefficient by multiple linear regression for age, BMI, cancer stage at diagnosis, duration since diagnosis, education level, working status, waking hours, sedentary time and MVPA. ^d^ Adjusted regression coefficient by multiple linear regression for age, BMI, cancer stage at diagnosis, duration since diagnosis, education level, working status, waking hours and sedentary time. * Significant *p*-value (< 0.05) for unadjusted variables. ** Significant *p*-value (< 0.05) for adjusted variables. *MVPA* Moderate to vigorous physical activity, *EE* Energy expenditure (Metabolic Equivalent of Task hours)

After adjusting for confounding factors, physical functioning (*p* = 0.007), role functioning (*p* = 0.010), and cognitive functioning (*p* = 0.023) were the domains of functional scores in EORTC QLQ-C30 that were negatively associated with sedentary behaviour among breast cancer survivors (Supplementary Material). These results indicated that an increased time spent on sedentary behaviour would significantly reduce most of the domains of the functional scores. However, only body image was significantly associated with prolonged sedentary bouts (*p* = 0.038) after adjusted for various confounders. In addition, the result showed that increased time spent in prolonged sedentary bouts significantly increased the body image among breast cancer survivors.

## Discussion

This study set out to determine the relationship of objectively measured physical activity and sedentary behaviour with HRQoL among breast cancer survivors in the East Coast of Peninsular Malaysia. Most of the previously published studies on the relationship between physical activity, sedentary behaviour, and HRQoL among breast cancer survivors relied on the subjective measurement of physical activity and sedentary behaviour [4, 9, 12]. It has been demonstrated that the use of objective measurement on physical activity and sedentary behaviour may reduce the risk of measurement error and misclassification [14]. To date, most studies on objectively measured physical activity or sedentary behaviour with HRQoL among breast cancer survivors were conducted in the United States [2]. One relevant study in Asia examined physical activity and HRQoL among breast cancer survivors but it applied self-reported measures of physical activity [9]. Therefore, this study would be able to fill the literature gap in terms of the relationship between objectively measured physical activity and sedentary behaviour and HRQoL among Malaysian breast cancer survivors.

Taken together, the results from previously published studies proposed an association between physical activity and HRQoL among breast cancer survivors. It has been reported that scores of fatigue and pain were lower with increased physical activity level after diagnosis [9]. The Women’s Healthy Eating and Living (WHEL) study conducted in the USA also found that breast cancer survivors who met physical activity guideline had a significantly higher overall HRQoL [24]. Similar findings on an association between increased physical activity and improved in HRQoL among breast cancer survivors were also reported in several other randomised control trials namely the LIVESTRONG at the YMCA Exercise Program [12], Better Exercise Adherence after Treatment for Cancer (BEAT) [25], and Exercise and Nutrition Enhance Recovery and Good Health for You (ENERGY) [26].

The findings of this study supported the work of other similar studies that linked physical activity and HRQoL. The results showed that MVPA was significantly associated with HRQoL after adjusted for age, BMI, cancer stage at diagnosis, education level, working status, waking hours, and sedentary time. Rossi et al., [27] studied the effect of exercise on HRQoL among cancer survivors and reported that vigorous physical activity might be more beneficial than low-intensity physical activity. However, there is only a limited number of studies comparing the effect of various physical activity intensity on HRQoL among cancer survivors. Thus, the association between physical activity intensity and HRQoL remains unclear [28]. In a study among 358 breast cancer survivors, a significant relationship between physical activity and HRQoL was observed whereby an increase in MVPA significantly improved the physical well-being besides reducing fatigue [14]. Additionally, in a study by Kim et al., [29] involving 5359 adults from the United States National Health and Nutrition Examination Survey, it was shown that increasing the level of physical activity might be a convenient strategy to improve HRQoL.

In addition, this study indicated an inverse relationship between objectively measured sedentary behaviour with HRQoL. After controlling for various confounders (age, BMI, cancer stage, duration since diagnosis, education level, working status, waking hours, and MVPA), sedentary behaviour (sitting or lying) was negatively associated with functional scores. Increased time spent on sedentary behaviour was associated with lower functional scores, namely physical functioning, role functioning, and cognitive functioning among breast cancer survivors. This is an important finding because improved functioning was reported to be associated with increased independence and reduced rate of adverse health outcomes [30]. It has also been suggested that improved functional status was the main goal in the therapy of cancer survivors [31]. Moreover, it is biologically plausible that sedentary time may affect HRQoL. The result of the present study was in line with a study by Lynch et al., [32] which found that colorectal cancer survivors who spent less time spent on sedentary behaviour had a significantly higher HRQoL compared to survivors who spent a longer time on sedentary behaviour. This study also reported a similar result as a previous study in which a decrease in sedentary behaviour was associated with improvements in physical functioning and role functioning [33]. Additionally, another study found that reduced daily sedentary behaviour was associated with improved HRQoL. It was reported that fatigue was significantly associated with daily minutes of sitting among breast cancer survivors [34]. However, George et al., [35] found that sedentary time was not independently related to long-term breast cancer survivors’ HRQoL or fatigue. Similarly, Vallance et al., [36] who investigated the association of objectively assessed physical activity and sedentary time with HRQoL among colon cancer survivors also reported no association between sedentary time and HRQoL.

Contrary to the popular beliefs, this study reported an association between increased time spent on prolonged sedentary bouts and improved body image. Body image has been shown to reflect a direct personal insight and self-evaluation of one’s physical appearance [37]. It has been found to be closely linked to attractiveness, sexual functioning, identity, self-esteem, and social relationships [38]. A higher level of positive body image was found among breast cancer survivors in this study. This could be attributed to the fact that the majority of the survivors in this study were older (≥ 50 years old) with a high prevalence of suboptimal health behaviours [39]. Older women were less likely to overthink about their body appearance and sexual functioning. A previous study among breast cancer survivors in Singapore reported that younger women were more concerned of their body image [40]. Another possible explanation for this result might be the fact that the participants were primarily overweight and obese in this study. Older and overweight individuals were frequently found to be having prolonged sedentary behaviour for most of their waking hours in order to reduce fatigue and to have a quality resting time [41]. Moreover, the majority of them were diagnosed more than 5 years ago. According to a previous study, body image commonly improved after 3 years of diagnosis [38]. Improved body image which generally improved their overall HRQoL seems might not motivate them to reduce their prolonged sedentary behaviour. As a result, they might not be motivated to reduce their prolonged sedentary behaviour to improve their HRQoL.

In recent years, the role of physical activity and the effects of sedentary behaviour on HRQoL among breast cancer survivors is generating a lot of attention in the scientific community. However, there is still a lack of data on the objective assessment of physical activity and sedentary behaviour among breast cancer survivors, especially in Asia. To the best of our knowledge, this is the first study that elucidated the relationship of objectively measured physical activity and sedentary behaviour with HRQoL among breast cancer survivors in the Asia region. The device used in this study, ActivPAL3™ microdevice, is able to distinguish the various positions of the wearer. It is also the most accurate device to assess steps cadence as it has a high degree of accuracy and reliability [42]. Step cadence is important in this study since it is used to distinguish between the low and high intensity physical activity. Furthermore, this study applied valid and reliable instruments to assess HRQoL including EORTC QLQ-C30 and EORTC QLQ-BR23, both of which have been proven to be suitable in assessing HRQoL among breast cancer patients [40]. These findings could provide more accurate guidance towards specific intervention programs tailored for breast cancer survivors to improve their survivorship.

There are several limitations to this study. Firstly, the cross-sectional study design employed cannot establish a causal relationship. Secondly, this study might not represent all breast cancer survivors in the East Coast of Peninsular Malaysia as it only included two main referral hospital in the region. Moreover, the participants who consented might have a higher interest in healthy lifestyle being more health-conscious and positive towards their disease than those who refused to take part. Hence, the sampled population might represent a higher percentage of participants with better MVPA and HRQoL. This study was also constrained by the limited sample size and high number of statistical tests (without adjustment for multiple testing). Finally, although the objectively measured physical activity used in the study was better than subjective measurements, it also carried certain limitations in terms of the inability of ActivPAL3™ microdevice to capture an accurate workload for certain activities such as swimming, biking, or weight training.

## Conclusion

In summary, engagement in MVPA was significantly related to HRQoL among Malaysian breast cancer survivors. Moreover, sedentary behaviour was shown to have a significant relationship with HRQoL. Higher sedentary behaviour may reduce functioning among breast cancer survivors. The study contributed additional evidence in this study area by indicating that increased time in MVPA was associated with improved HRQoL while sedentary behaviour was associated with poorer HRQoL among breast cancer survivors. However, prolonged sedentary bouts were shown to be associated with a better body image that might subsequently improve HRQoL. This warrant further prospective and intervention studies to confirm the relationship of objectively measured physical activity with sedentary behaviour and HRQoL among Malaysian breast cancer survivors. It also provides valuable information to the stakeholders such as clinicians and public health practitioners to advise breast cancer patients to increase MVPA and reduce time spent on sedentary behaviour in order to improve their HRQoL.

## Supplementary information

**Additional file 1:** Supplementary Material.

## Data Availability

The dataset used and/or analysed during the current study are available from the corresponding author on reasonable request.

## Abbreviations

BMI: Body Mass Index
BEAT: Better Exercise Adherence after Treatment for Cancer
EORTC: European Organization for Research and Treatment
ENERGY: Exercise and Nutrition Enhance Recovery and Good Health for You
HRQoL: Health-related quality of life
MREC: Medical Research and Ethics Committee
MVPA: Moderate-to-vigorous physical activity
WHEL: Women’s Healthy Eating and Living
