# Correction to: Relationship of objectively measured physical activity and sedentary behaviour with health-related quality of life among breast cancer survivors

**DOI:** 10.1186/s12955-020-01513-x

**Published:** 2020-08-06

**Authors:** Ali Nurnazahiah, Mohd Razif Shahril, Zakarai Nor Syamimi, Aryati Ahmad, Suhaina Sulaiman, Pei Lin Lua

**Affiliations:** 1 Faculty of Health Sciences, Universiti Sultan Zainal Abidin, Gong BadakCampus, 21300 Kuala Nerus, Terengganu Malaysia; 2 Centre for HealthyAgeing and Wellness (H-CARE), Faculty of Health Sciences, UniversitiKebangsaan Malaysia, Jalan Raja Muda Abdul Aziz, 50300 Kuala Lumpur, Malaysia; 3 Faculty of Pharmacy, Universiti Sultan Zainal Abidin, Besut Campus, 22200 Besut, Terengganu Malaysia

**Correction to: Health Qual Life Outcomes 18, 222 (2020)**

**https://doi.org/10.1186/s12955-020-01478-x**

The original article [1] contained an error whereby co-author, Aryati Ahmad’s name was displayed incorrectly. This has since been corrected.
