# Supplementary material for: Relationship of objectively measured physical activity and sedentary behaviour with health-related quality of life among breast cancer survivors
Source: Health Qual Life Outcomes. 2020 Jul 10;18:222. doi: 10.1186/s12955-020-01478-x (PMC7350582; doi:10.1186/s12955-020-01478-x)
Supplement: Supplementary file 1 — Additional file 1: Supplementary Material. [file 12955_2020_1478_MOESM1_ESM.docx]

**Supplementary Material**

Supplementary Table A: Domains of Functional Scores (EORTC QLQ-C30) Associated with Sedentary Time (Sitting/Lying)

| **Sitting/lying** | | |
| --- | --- | --- |
| **EORTC QLQ-C30 Domains** | **Regression Coefficient (β) (95% CI)** | **p-value** |
| Physical functioning |  |  |
| Unadjustedª | -1.860 (-3.107, -0.613) | 0.004* |
| Adjusted^b^ | -1.968 (-3.381, -0.554) | **0.007**** |
| Role functioning |  |  |
| Unadjustedª | -1.765 (-3.112, -0.419) | 0.011* |
| Adjusted^b^ | -2.037 (-3.573, -0.502) | **0.010**** |
| Emotional functioning |  |  |
| Unadjustedª | -0.387 (-2.391, 1.617) | 0.702 |
| Adjusted^b^ | -0.022 (-2.369, 2.326) | 0.985 |
| Cognitive functioning |  |  |
| Unadjustedª | -3.358 (-5.705, -1.011) | 0.006* |
| Adjusted^b^ | -3.065 (-5.693, -0.438) | **0.023**** |
| Social functioning |  |  |
| Unadjustedª | -1.966 (-3.475, -0.458) | 0.011* |
| Adjusted^b^ | -1.448 (-3.168, -0.271) | 0.097 |

ª Crude regression coefficient by simple linear regression, ^b^ Adjusted regression coefficient by multiple linear regression for age, BMI, cancer stage at diagnosis, duration since diagnosis, education level and working status.  * Significant *p*-value (<0.05) for unadjusted variables. ** Significant *p*-value (<0.05) for adjusted variables.

Supplementary Table B: Domains of Functional Scores (EORTC QLQ-BR23) Associated with Prolonged Sedentary Bouts

| **Prolonged sedentary bouts** | | |
| --- | --- | --- |
| **EORTC QLQ-BR23 Domains** | **Regression Coefficient (β) (95% CI)** | **p-value** |
| Body image |  |  |
| Unadjustedª | 0.520 (-0.352, 1.393) | 0.239 |
| Adjusted^b^ | 1.332 (0.072, 2.591) | **0.038**** |
| Sexual functioning |  |  |
| Unadjustedª | -2.600 (-4.108, -1.092) | 0.001* |
| Adjusted^b^ | -1.989 (-4.163, 0.184) | 0.072 |
| Sexual enjoyment |  |  |
| Unadjustedª | -2.545 (-4.240, -0.850) | 0.004* |
| Adjusted^b^ | -1.940 (-4.498, 0.618) | 0.135 |
| Future perspective |  |  |
| Unadjustedª | 0.505 (-1.243, 2.253) | 0.567 |
| Adjusted^b^ | 1.625 (-0.877, 4.127) | 0.200 |

ª Crude regression coefficient by simple linear regression, ^b^ Adjusted regression coefficient by multiple linear regression for age, BMI, cancer stage at diagnosis, duration since diagnosis, education level and working status. * Significant *p*-value (<0.05) for unadjusted variables. ** Significant *p*-value (<0.05) for adjusted variables.
